# Relationship between production of acute-phase proteins and strength of inflammatory stimulation in rats

**DOI:** 10.1258/la.2011.010112

**Published:** 2011-07

**Authors:** Takashi Kuribayashi, Misaki Tomizawa, Tetsurou Seita, Kazutoshi Tagata, Shizuo Yamamoto

**Affiliations:** Laboratory of Immunology, School of Life and Environmental Science, Azabu University, 1-17-71 Fuchinobe, Chuo-ku, Sagamihara, Kanagawa 252-5201, Japan

**Keywords:** *α*2M, AAG, IL-6, CINC-1, rats

## Abstract

The relationship between intensity of inflammatory stimulation and production of *α*_2_-macroglobulin (*α*2M) and *α*_1_-acid glycoprotein (AAG) in rats was investigated. Sprague-Dawley rats were injected with turpentine oil at doses of 0.05, 0.2 or 0.4 mL/rat. Serum levels of *α*2M, interleukin (IL)-6 and cytokine-induced neutrophil chemoattractant-1 (CINC-1) were measured by enzyme-linked immunosorbent assay, and AAG was measured by single radial immunodiffusion. Peak serum levels of *α*2M and AAG in rats injected at 0.05 mL/rat were significantly lower than those at 0.2 or 0.4 mL/rat. However, no significant differences were observed for peak serum levels of these acute-phase proteins between 0.2 and 0.4 mL/rat. Furthermore, peak serum levels of IL-6 and CINC-1 in rats injected at 0.05 mL/rat were significantly lower than those at 0.2 or 0.4 mL/rat. Thus, the production of these acute-phase proteins has upper limits, even under increased strength of inflammatory stimulation in rats injected with turpentine oil.

Acute-phase protein levels increase during acute inflammation, e.g. bacterial infection, trauma and surgical treatment.^1–3^ C-reactive protein is a typical acute-phase protein in humans and dogs,^4–7^ while *α*_2_-macroglobulin (*α*2M) is a typical acute-phase protein in rats.^8–10^ *α*2M levels increase in rats subjected to acute inflammation by inoculation with microorganisms, injection of turpentine oil or after surgical treatment.^9^ *α*_1_-acid glycoprotein (AAG) contributes to protein binding with anionic drug substances, and also increases following inflammatory stimulation.^11,12^

Peak levels of *α*2M differ after injection of turpentine oil, inoculation with microorganisms or surgical treatment.^8,9^ Furthermore, peak levels of AAG also differ after injection of turpentine oil, inoculation with microorganisms and gastric bleeding by injection of indomethacin.^12^ Peak levels of these acute-phase proteins are thus presumed to differ with the magnitude of inflammatory stimulation in rats. *α*2M and AAG are considered to be typical inflammatory markers in rats. However, there has been little basic research on these proteins, and little is known about the differences in production based on the magnitude of stimulation. Thus, degree of inflammation cannot be estimated based on production of *α*2M or AAG.

In the present study, the changes in serum levels of *α*2M and AAG in rats subjected to various dosages of turpentine oil to induce acute inflammation were investigated in an effort to clarify whether the production of acute-phase proteins varies with the magnitude of inflammatory stimulation.

## Materials and methods

### Animals

Eighteen nine-week-old Sprague-Dawley rats were purchased from Charles River, Inc (Yokohama, Japan), and were used in the present study. Rats were kept in isolators at a temperature of 23 ± 2°C, and a relative humidity of 55 ± 10%, on a 12/12 h dark/light cycle (06:00–18:00) with air exchanged 12 times or more per hour. Rats were fed MF (Oriental Yeast Co, Ltd, Tokyo, Japan), and were allowed free access to water.

All experiments were approved by the Institutional Review Board of Azabu University and conducted in accordance with the Institute's Animal Experimentation Guidelines (Japanese Association for Laboratory Animal Science, JALAS, 1987).

### Animal experiment design

Rats were divided into three groups (*n* = 6 for each group). Turpentine oil (Wako Pure Chemical Industries, Ltd, Osaka, Japan) was injected intramuscularly into rats at 0.05, 0.2 or 0.4 mL/rat. Acute-phase protein levels are known to increase after intramuscular injection of turpentine oil,^8,9,13–15^ which has been used to induce acute inflammation in several studies, primarily because turpentine oil is able to induce constant acute inflammation, as compared with infection by microorganisms or other chemical compounds.^8,9^ Turpentine oil was thus used to induce acute inflammation in this study. Blood (0.3 mL) was collected before injection and at 6, 12, 24, 48, 72 and 96 h after administration. Blood was collected from the venae cervicalis superficialis using a syringe under mild anaesthesia with pentobarbital (Kyoritsu Seiyaku Corporation, Tokyo, Japan). Pentobarbital was intravenously administered at small doses (4 mg/kg) in order to ensure only short-term effects. Sera were obtained by centrifugation at 1600 ***g*** for 15 min, and were stored at −80°C until analysis.

### Measurement of *α*2M and AAG

Serum levels of *α*2M were measured by enzyme-linked immunosorbent assay (ELISA) according to the procedure of Honjo *et al.* ^16^ Serum levels of AAG were measured by single radial immunodiffusion using a commercial kit (Institute for Metabolic Ecosystem Co, Ltd, Miyagi, Japan).

### Measurement of interleukin-6 and cytokine-induced neutrophil chemoattractant-1

Serum levels of interleukin-6 (IL-6) and cytokine-induced neutrophil chemoattractant (CINC-1) were measured by ELISA using commercial kits. Commercial ELISA kits were purchased from BioSource International, Inc (Camarillo, CA, USA) for IL-6 and from Panapharm Laboratories Co, Ltd (Kumamoto, Japan) for CINC-1.

### Statistics

All values are expressed as means ± SD (*n* = 6). Analysis of significance between variables was performed using unpaired Student's *t*-test. *P* values of <0.05 were considered to be statistically significant.

## Results

**Figure 1:**
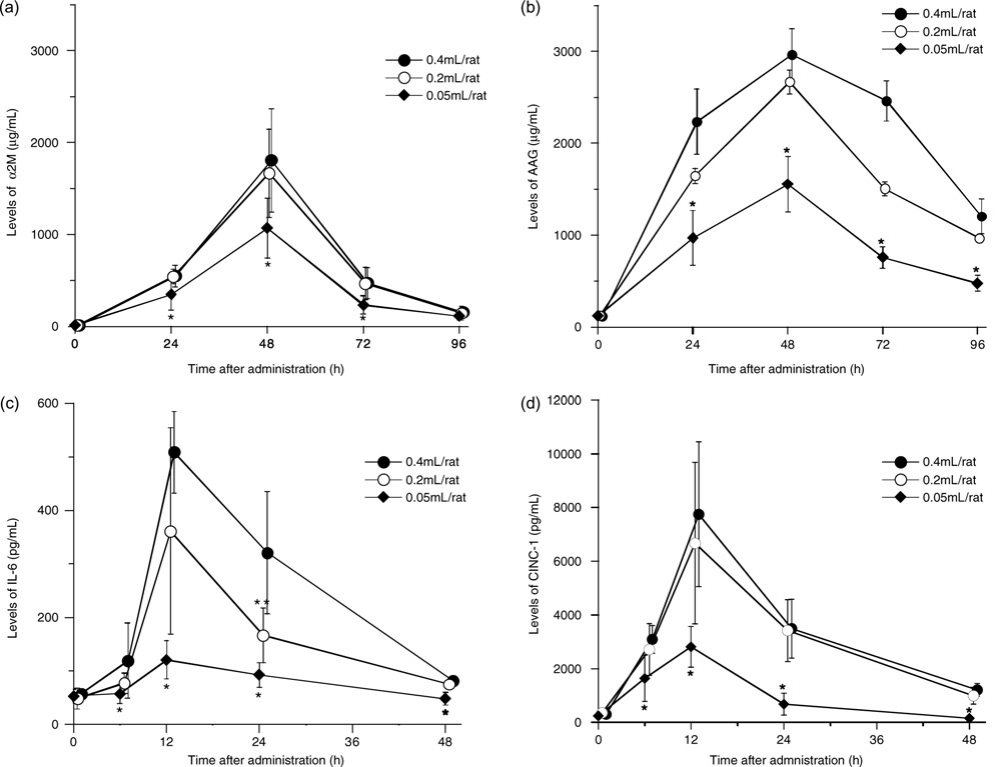
Changes in serum levels of acute-phase proteins and cytokines in rats injected with turpentine oil at 0.05, 0.2 or 0.4 mL/rat. (a) *α*_2_-macroglobulin (*α*2M), (b) *α*_1_-acid glycoprotein (AAG), (c) interleukin-6 (IL-6) and (d) cytokine-induced neutrophil chemoattractant-1 (CINC-1). Data are expressed as means ± SD (*n* = 6). *P < 0.05, significantly different versus 0.2 or 0.4 mL/rat injection group, as analysed by Student's *t*-test. **P < 0.05, significantly different versus 0.4 mL/rat injection group, as analysed by Student's *t*-test

Changes in serum levels of *α*2M, AAG, IL-6 and CINC-1 are shown in Figure 1. Peak levels of *α*2M were observed at 48 h after injection of 0.05, 0.2 or 0.4 mL/rat. Peak *α*2M levels were 1071.2 ± 324.4 µg/mL in the 0.05 mL/rat injection group, 1667.4 ± 478.7 µg/mL in the 0.2 mL/rat injection group and 1808.0 ± 563.5 µg/mL in the 0.4 mL/rat injection group. Peak levels in the 0.05 mL/rat injection group were significantly lower than those in the 0.2 and 0.4 mL/rat injection groups. However, no significant differences were observed between the 0.2 and 0.4 mL/rat injection groups.

Serum levels of AAG also peaked at 48 h after injection in all groups. Peaks for AAG were 1554.8 ± 302.8 µg/mL in the 0.05 mL/rat injection group, 2667.6 ± 451.1 µg/mL in the 0.2 mL/rat injection group and 2962.8 ± 285.0 µg/mL in the 0.4 mL/rat injection group. Peak levels of AAG in the 0.05 mL/rat injection group were significantly lower than those in the 0.2 and 0.4 mL/rat injection groups.

Peak serum levels of IL-6 and CINC-1 were observed at 12 h after injection in all injection groups. Peak levels of IL-6 were 120.5 ± 36.0 pg/mL in the 0.05 mL/rat injection group, 361.0 ± 192.9 pg/mL in the 0.2 mL/rat injection group and 508.6 ± 76.2 pg/mL in the 0.4 mL/rat injection group. Peak levels in the 0.05 mL/rat injection group were significantly lower than in the 0.2 and 0.4 mL/rat injection groups. No significant differences were observed between the 0.2 and the 0.4 mL/rat injection groups, except at 24 h after injection.

Peaks for CINC-1 were 2812.3 ± 755.8 pg/mL in the 0.05 mL/rat injection group, 6734.8 ± 2898.2 pg/mL in the 0.2 mL/rat injection group and 7449.8 ± 2698.5 pg/mL in the 0.4 mL/rat injection group. Peaks levels in the 0.05 mL/rat injection group were significantly lower than those in the 0.2 and 0.4 mL/rat injection groups. No significant differences were observed between the 0.2 and 0.4 mL/rat injection groups.

## Discussion

Serum levels of the typical acute-phase proteins *α*2M and AAG in rats after inflammatory stimulation with various doses of turpentine oil were examined in an effort to clarify the differences in production during various degrees of inflammatory stimulation. Serum levels of *α*2M and AAG in rats injected with 0.05 mL/rat were significantly lower than those injected with 0.2 and 0.4 mL/rat; however, no significant differences were observed between the 0.2 and 0.4 mL/rat injection groups. These results suggest that production of *α*2M and AAG increases in proportion with turpentine oil dose. Production of *α*2M and AAG was similar above 0.2 mL/rat; however, limited production was observed. Thus, it was necessary to carefully evaluate the degree of inflammation based on the levels of these acute-phase proteins.

IL-6 is known to regulate the synthesis of acute-phase proteins,^17–22^ while IL-8 regulates the production of acute-phase proteins in humans.^23,24^ However, IL-8 has not been observed in rats, and CINC-1 is considered to be the counterpart of human IL-8.^25–28^ On the other hand, serum levels of seven cytokines (IL-1, IL-2, IL-4, IL-6, CINC-1, IL-10 and interferon-*γ*) in rats injected with turpentine oil have been investigated, and only serum levels of IL-6 and CINC-1 were elevated prior to increases in *α*2M.^9^ In contrast, levels of the cytokines IL-1, IL-2, IL-4, IL-10 and interferon-*γ* did not change.^9^ IL-6 and CINC-1 also increased prior to AAG in rats injected with turpentine oil.^12^ Furthermore, the changes in serum levels of *α*2M and AAG after repeated injection of turpentine oil at doses of 0.05 and 0.4 mL/rat showed a similar pattern as with single stimulation, and peak serum levels in rats injected with 0.05 and 0.4 mL/rat were largely comparable. This phenomenon was thought to be caused by differences in the production of IL-6 and CINC-1 in the two groups.^29^ Thus, IL-6 and CINC-1 apparently contribute to the production of *α*2M and AAG in rats.^16^ In the present study, levels of IL-6 and CINC-1 in rats injected with 0.05 mL were also significantly lower than those injected with 0.2 and 0.4 mL/rat. The levels of both cytokines did not differ significantly between the 0.2 and the 0.4 mL/rat injection groups. The four parameters evaluated in the present study showed similar patterns. The significant differences in production of *α*2M and AAG independent of the turpentine oil dosage are considered to be attributable to significant differences in the production of IL-6 and CINC-1. These results thus suggest that IL-6 and CINC-1 regulate the production of *α*2M and AAG.

In summary, the production of *α*2M and AAG has an upper limit, irrespective of the magnitude of stimulation. Production of *α*2M and AAG showed similar trends to those of IL-6 and CINC-1; thus, the kinetics of these cytokines influenced the production of *α*2M and AAG.
